# Self-renewal and differentiation capabilities are variable between human embryonic stem cell lines I3, I6 and BG01V

**DOI:** 10.1186/1471-2121-10-44

**Published:** 2009-06-05

**Authors:** Tahereh Tavakoli, Xiangru Xu, Eric Derby, Yevgeniya Serebryakova, Yvonne Reid, Mahendra S Rao, Mark P Mattson, Wu Ma

**Affiliations:** 1 Stem Cell Center, Developmental Biology, American Type Culture Collection (ATCC), Manassas, VA, USA; 2 Laboratory of Neurosciences, National Institute on Aging, Intramural Research Program, National Institutes of Health, Baltimore, MD, USA; 3 Stem Cells and Regenerative Medicine (Research), Invitrogen, Carlsbad, CA, USA; 4 Current address: iZumi Bio Inc., 951 Gateway Boulevard, South San Francisco, CA 94080, USA

## Abstract

**Background:**

A unique and essential property of embryonic stem cells is the ability to self-renew and differentiate into multiple cell lineages. However, the possible differences in proliferation and differentiation capabilities among independently-derived human embryonic stem cells (hESCs) are not well known because of insufficient characterization. To address this question, a side-by-side comparison of 1) the ability to maintain an undifferentiated state and to self-renew under standard conditions; 2) the ability to spontaneously differentiate into three primary embryonic germ lineages in differentiating embryoid bodies; and 3) the responses to directed neural differentiation was made between three NIH registered hES cell lines I3 (TE03), I6 (TE06) and BG01V. Lines I3 and I6 possess normal XX and a normal XY karyotype while BG01V is a variant cell line with an abnormal karyotype derived from the karyotypically normal cell line BG01.

**Results:**

Using immunocytochemistry, flow cytometry, qRT-PCR and MPSS, we found that all three cell lines actively proliferated and expressed similar "stemness" markers including transcription factors POU5F1/Oct3/4 and NANOG, glycolipids SSEA4 and TRA-1-81, and alkaline phosphatase activity. All cell lines differentiated into three embryonic germ lineages in embryoid bodies and into neural cell lineages when cultured in neural differentiation medium. However, a profound variation in colony morphology, growth rate, BrdU incorporation, and relative abundance of gene expression in undifferentiated and differentiated states of the cell lines was observed. Undifferentiated I3 cells grew significantly slower but their differentiation potential was greater than I6 and BG01V. Under the same neural differentiation-promoting conditions, the ability of each cell line to differentiate into neural progenitors varied.

**Conclusion:**

Our comparative analysis provides further evidence for similarities and differences between three hESC lines in self-renewal, and spontaneous and directed differentiation. These differences may be associated with inherited variation in the sex, stage, quality and genetic background of embryos used for hESC line derivation, and/or changes acquired during passaging in culture.

## Background

Human embryonic stem cells (hESCs) possess the ability to self-renew in an undifferentiated state in culture while retaining the ability to differentiate into all of the cell types in the human body. These unique capabilities make hESCs a renewable source of a wide range of cell types for potential use in research and cell-based drug screening and therapies for many diseases. These cells have been in high demand for use in basic and applied biomedical research. As of January 1, 2006, at least 414 human ES cell lines have been derived worldwide [1]. Large numbers of cell lines with genetic diversity are necessary to cover the vast spectrum of HLA isotypes to avoid transplant rejection [2,3]. However, many of these cell lines are not fully characterized and differences among these cell lines are uncertain [1], although recent studies have revealed similarities and differences among individually developed human embryonic stem cell lines [3-12].

The comparison of the unique properties and behavior of each individually derived cell line is critical in identifying the safe and efficacious lines for research and therapeutic use [3,13]. It is also essential to understand how the inherited variation in the sex, stage, quality and genetic background of embryos, as well as environmental influences such as derivation methods and passage procedures can affect the ability of hES cell lines to self-renew and differentiate. Directly comparing hES cell lines is challenging since all the genetic, environmental and methodological variables complicate the assessments. Previous studies have attempted setting up a core set of standard assays to characterize the status of "stemness" and pluripotency [14] and to define a reasonable set of markers that would serve as reliable indicators for self-renewal and differentiation of hESCs [10,12]. In the present study, a side-by-side comparison of the ability to maintain an undifferentiated state and to self-renew under standard conditions, the ability to spontaneously differentiate into cell types of three germ layers in embryonic bodies, and directed differentiation under neural differentiation-promoting conditions was made between three NIH registered hESC lines I3, I6 and BG01V. I3 (NIH Registry Name TE03) and I6 (NIH Registry Name TE06) which were derived using rabbit anti-human whole antiserum with a normal XX and a normal XY karyotype respectively [15]; BG01V contains known chromosomal aberrations (XXY, +12 and +17) possesses characteristics similar to its normal parental line BG01 [16,17]. The hESC lines I3, I6 and BG01V have been extensively characterized and tested in our laboratory for potential reference standard cell lines, because these three lines represent consensus standard human ES cell lines and a karyotypically abnormal human ES cell variant respectively.

Immunocytochemistry, flow cytometry, quantitative RT-PCR and MPSS were used to assess the self-renewal and differentiation capabilities. We found that all three cell lines actively proliferated and expressed similar "stemness" and pluripotency markers and alkaline phosphatase activity. All the cell lines differentiated into phenotypes representing ectoderm, endoderm, and mesoderm and were directed into neural cell lineages *in vitro*. However, the significant differences were observed in growth rate, BrdU incorporation, relative abundance of pluripotency marker expression and the ability to differentiate. These differences between the cell lines may depend on a combination of genetic, environmental and methodological factors [3], implicating the importance of establishing standard protocols for hESC derivation and culture.

## Methods

### Cell Culture

Human embryonic stem cell lines I3, I6, and BG01V used in this study were cultured on mitomycin C-treated mouse embryonic fibroblasts CF-1 (ATCC, SCRC-1040.2; ). Cells were cultured at 37°C, in a 5% CO_2 _atmosphere, in the ES medium of Dulbecco's modified Eagle's medium (D-MEM)/F12 (ATCC 30-2006)80%, supplemented with 2.0 mM L-alanyl-L-glutamine (ATCC 30-2115), 0.1 mM non-essential amino acids (ATCC 30-2116), 0.1 mM 2-mercaptoethanol (Sigma Catalog No. M-7522) and 4 ng/ml basic fibroblast growth factor (bFGF; R & D Systems Catalog No. 233-FB), 5%; Knockout serum replacement (Invitrogen Catalog No. 10828), 15%; fetal bovine serum (ATCC SCRR-30-2020), penicillin (100 I.U./mL) and streptomycin (100 μg/mL) (ATCC 30-2300). An additional 4 ng/ml of bFGF was added in the first 24 hours after thawing the cells. Daily medium changes began after the first 48 hours in culture. The BG01V colony formation was visible within 2–3 days and the other two cell line's colony formation was observed in 3–4 days. Cells were passaged every 4–5 days using collagenase IV (200 Units/mL) (Invitrogen Corporation) for BG01V, I6 was passaged every 6–7 days and I3 was passaged every 7–8 days.

### Embryoid body formation

hESCs in culture were removed from feeder cells using collagenase IV (200 Units/mL) (Invitrogen Corporation; ). hESC clusters were transferred to 10 × 10-cm ultra-low-attachment dishes (corning; ) and cultured in medium D-MEM/F12 (80%) (ATCC 30-2006) supplemented with ES-Qualified FBS (15%) (ATCC SCRC-30-2020), knockout serum replacement (KSR) (5%) (Invitrogen Corporation), L-alanyl-L-glutamine (2.0 mM) (ATCC 30-2115), non-essential amino acids (1×) (ATCC 30-2116), β-mercaptoethanol (0.1 mM) (Invitrogen Corporation), penicillin (100 I.U./mL)/streptomycin (100 μg/mL) (ATCC 30-2300). The medium was changed every second day. To evaluate the growth rates of EBs, phase-contrast photographs of EBs were taken and the total areas of EBs were measured using Scion Image. The percent increase in total areas of the cell spheroids was compared between different cell lines. Data were calculated as mean ± S.E.M. of at least 3 separate cultures. The statistical significance was determined using the Student's *t*-test with p < 0.05 considered significant.

### Directed neural differentiation of hESCs

The directed neural differentiation method was described previously [18]. Briefly, colonies of the three hESC lines I3, I6, BG01V were removed from MEF feeders and dissociated into small clumps by incubating with collagenase IV (200 Units/ml) (Invitrogen Carlsbad, CA; ) at 37°C for 35 minutes. The hESC clumps were pelleted and cultured in suspension in low attachment dishes with hESC medium without bFGF for 5 days (the end of this stage is considered as 5 days of differentiation). hESC grew into floating aggregates or embryoid bodies (EBs). The neuroectodermal induction began with EBs transferred into the neural differentiation medium (NDM) that consisted of two parts of a modified Eagle's medium (ATCC 30-2002; ), one part F12k- (ATCC 30-2004), 1× N-2 supplement (Gibco Catalog No. 317740; ), 0.1 mM non-essential amino acids (ATCC 30-2116), penicillin (100 IU/ml)/streptomycin (100 μg/ml) (ATCC 30-2300) and 5 ng/ml bFGF (R& D Systems Catalog No. 233-FB) for 10 days. At days 15–17 of differentiation, EBs were plated on PDL/laminin substrate-coated 35 mm dishes (corning; ). Although some neural rosettes were formed in floating embryoid bodies (EBs), increased rosettes were visualized after plating of the EBs on substrates. Neuroectodermal cells in rosettes were further differentiated into neural progenitors and their progeny on PDL/laminin substrates.

### Growth curve

To compare the growth rate between I3, I6 and BG01V, all three hES cells were plated into 6 well plates containing a feeder layer of mitomycin C-treated fibroblast (MEF). The cells were cultured at 37°C in a 5% CO_2 _atomosphere. Basic fibroblast growth factor (4 ng/ml) was added to each cell culture after the first 24 hours. The medium was changed daily after 48 hours. The cells from three separate wells were harvested using a 0.25% (w/v) trypsin/0.53 mM EDTA solution (ATCC cat # 30-2101) each day. The cell counts were performed using Cedex Analysis System, Innovatis. Data were calculated as mean ± S.E.M. of at least 3 separate cultures. The statistical significance was determined using the Student's *t*-test with p < 0.05 considered significant.

### Bromodeoxyuridine (BrdU) incorporation and counterstaining with propidium iodide (PI)

To monitor cell proliferation within colonies of hES cells, bromodeoxyuridine (BrdU) incorporation with 5-bromo-2-deoxy-uridine Labeling and Detection Kit I (Roche, Indianapolis, IN; ) was used as described previously [18]. Briefly, cultures were exposed to 20 μM BrdU for 4 hours and then fixed with 70% alcohol containing 50 mM glycine at PH 2.0. After rinsing with the kit wash buffer, cells were incubated overnight with mouse anti-BrdU (1:1000) followed by incubation with FITC-conjugated donkey anti-mouse IgG (1:50) (Jackson Immunological Research, West Grove, PA) for 45 min. Some cultures that were not exposed to BrdU were used as negative controls which showed no immunoreactivity, demonstrating the specificity of BrdU antibody. In order to quantify the cell proliferation rate, cell nuclei were counterstained by the addition of 5 μg/ml propidium iodide (PI) for 10 min. PI+ and BrdU+ cells were examined and photographed with Nikon eclipse TE 300 microscope. The proliferation index was defined as the percentage of BrdU+ nuclei in the total number of PI+ cells. At least 5 labeled colonies were counted from each dish and three dishes were evaluated. Data were calculated as mean ± S.E.M. which were statistical significance determined by using the Student's *t*-test with p < 0.05 considered significant.

### Immunocytochemistry

For the immunostaining of undifferentiated hES colonies for "stemness" markers, undifferentiated hESCs were cultured on mitomycin C-treated feeder cells in 35 mm tissue culture dishes (Corning, Corning, NY, ). Colonies were rinsed twice before fixation with 4% paraformaldehyde (EMS, Hatfield, PA, ) in 1× PBS for 15 min at room temperature. Cells were permeabilized with 0.5% saponin (Sigma, St. Louis, MO, ) in PBS for 10 min. Primary antibodies against Oct3 (BD Biosciences, San Jose, CA, , 1:250), NANOG (1:100), and SSEA4 (1:100), and TRA-1-81 (1:50) (all from Millipore, Billerica, MA ) were incubated with colonies overnight at 4°C. The secondary antibodies used were either, Alexa Fluor 488 conjugated goat anti-mouse IgG (H+L), (Invitrogen/Molecular Probes, Eugene, OR, ; 1:50), or FITC-conjugated donkey anti-Mouse IgM (Jackson ImmunoResearch, West Grove, PA, , 1:50), or Alexa flour 488-conjugated rabbit anti-goat IgG1 (Invitrogen/Molecular Probes, Eugene, OR, ). Colonies were incubated with secondary antibodies for 45 min at room temperature.

Immunostaining of hESC-derived neural cells for Nestin and TuJ1 were performed as described previously [18]. Briefly, neural differentiation medium-treated EB were plated on PDL/laminin coated 35 mm Tissue Culture plates (Corning, Corning, NY, ). Differentiated cells were fixed with 4% paraformaldehyde and permeabilized in 0.5% saponin as described above. Primary antibodies used were rabbit anti-nestin, 1:200, mouse anti-tubulin clone TUJ-1, 1:300, chicken anti-SOX1, 1:200, (all from Millipore, Billerica, MA ). Secondary antibodies used were either rhodamine-conjugated donkey anti- rabbit IgG-(H+L) (Jackson Immunoresearch, West Grove, PA; ), Alexa Fluor 488 conjugated goat anti-mouse- IgG (H+L) (Molecular Probes, Eugene, Oregon; ) or FITC-conjugated donkey anti-chicken IgG (Millipore, Billerica, MA , 1:50). Cells were counterstained with the 4'-6-diamidino-2-phenylindole (DAPI) with dilution of 1:1000 (Sigma; ). Immunofluorescence signals were observed and photographed with a Nikon TE 300 epifluorescence microscope (Nikon, Inc. Melville, NY) equipped with a Qicam FAST1394 digital camera (Surrey, BC, Canada) and Openlab vs. 4.0.4 software .

To quantify the percentage of hESC-differentiated neural progenitors, cell counting was performed on cultures immunostained for nestin, together with nuclear DAPI counterstaining, in 35 mm culture dishes coated with different laminin substrates from at least three independent experiments. All data were expressed as mean ± SEM, and Student's *t *test was used for statistical evaluation. In all instances p < 0.05 was considered statistically significant.

### Alkaline Phosphatase staining

Endogenous alkaline phosphatase activity in BG01V, I3 and I6 cells was detected using the ELF^® ^97 Endogenous Alkaline Phosphatase Detection Kit (ATCC catalog # SCRR-3010) according to the manufacturer's instructions. Cells cultured in 6 well plates (Corning Life Sciences; ) were treated with 4% paraformaldehyde for 15 minutes at room temperature. The cells were washed with 1× PBS, treated with 0.2% Tween-20 for 10 minutes at room temperature and rinsed with 1× PBS. Fixed cells were then incubated with a filtered 1:20 dilution of the phosphatase substrate *in situ*, and the reaction was monitored using an epifluorescence microscope. The reaction was terminated using a stop solution consisting of PBS, 25 mM EDTA, 5 mM levamisole, pH 8.0. Cells were rinsed with PBS before mounting on glass microscope slides.

### qRT-PCR

Total RNA was isolated from three hESC lines I3, I6, and BG01V using an RNAeasy Plus Mini kit (Qiagen catalog NO 74134; ). The isolated RNAs were quantified using a RNA 6000 Nano Kit (Catalog NO 5067-1511). The integrity of RNA was checked on Agilent 2100 Bioanalyzer (Agilent Technologies;  part No G2940CA). Equal amounts of RNA (1 μg) was taken for all samples and reverse transcription was done using RT^2 ^First Strand kit from Superarray Biosciences (SuperArray, catalog No C-03; . The total volume of the reaction was 20 μL and was diluted to 100 μL. PCR reactions were performed using a ABI Fast 7900 using RT^2 ^Real-Time™ SYBR Green PCR master mix PA-012 and qRT-PCR primers from SuperArrray Biosciences. The total volume of the PCR reaction was 10 μL. The qRT-PCR Primers sets catalog numbers are 18srRNA PPH00073A, B-actin PPH05666A, POU5F1, Nanog, UTF1 (undifferentiated) PPH02394A, PPH02391A and PPH17032A, keratin C and NEFL (ectoderm) PPH-21369A and PPH02430A, alpha-globin and Beta-globin (Mesoderm) PPH09054A and PPH12971A, alpha-1AT PPH02413A, nestin and Musashi 1, SOX1 (Neural Progenitor) PPH02388A, PPH13090A and PPH02390A, MAP2, GAD-65 (Neural) PPH02419A, PPHo5950A, S100B and GFAP (astrocytes) PPH02408A and PPH02472A; . The thermocycler parameters were 95°C for 10 min, followed by 40 cycles of 95°C for 15 sec and 60°C for 1 min. Each gene for each sample was run in quadruplicate. Relative changes in gene expression were calculated using the ΔΔC_t _(threshold cycle) method. This method first subtracts the ct (Threshold cycle number) of the gene-avg ct of the two house keeping genes (18srRNA and ACTB) to normalize to the RNA amounts. Finally, the delta delta ct is calculated by subtracting the normalized average ct of the treated cells from the normalized average ct of the undifferentiated cells. Then this delta ct is raised to the negative power of 2 in order to calculate the fold change [19].

### Massively Parallel Signature Sequencing

Massively parallel signature sequencing (MPSS) was performed using 1–2 μg purified total RNA from each of the three human ESC lines (BG01V, I3 and I6) from undifferentiated cells (day 0) and cells at different stages of differentiation (days 7, 14 and 21); the presence and absence of ESC markers and markers of differentiation were evaluated. The quality of total RNAs was evaluated using an Agilent Bioanalyzer. mRNA isolation was processed according to the MPSS protocol as described previously [20] with some modification. In brief, the mRNA was reverse-transcribed, the cDNA synthesized and digested with *Dpn*II, then GEX adaptors ligated with *Dpn*II and amplified by PCR, the cDNA library was ready to sequence. The abundance for each signature was converted to transcripts per million (tpm) for the purpose of comparison between samples. Only reliable and annotatable signatures against updated human signature database were considered for further analysis.

To generate a complete, annotated human signature database, all the possible signatures from the human genome sequence, the human UniGene sequences, and human mitochondrion were extracted. Each virtual signature was ranked based on its position and orientation in the original sequence. The annotation database is established based on the virtual signatures, their classes and their corresponding genes so that each signature only has one corresponding annotation. The database is then used to annotate the data from the experiment.

## Results

### Different hESC lines exhibit different colony morphologies

**Figure 1:**
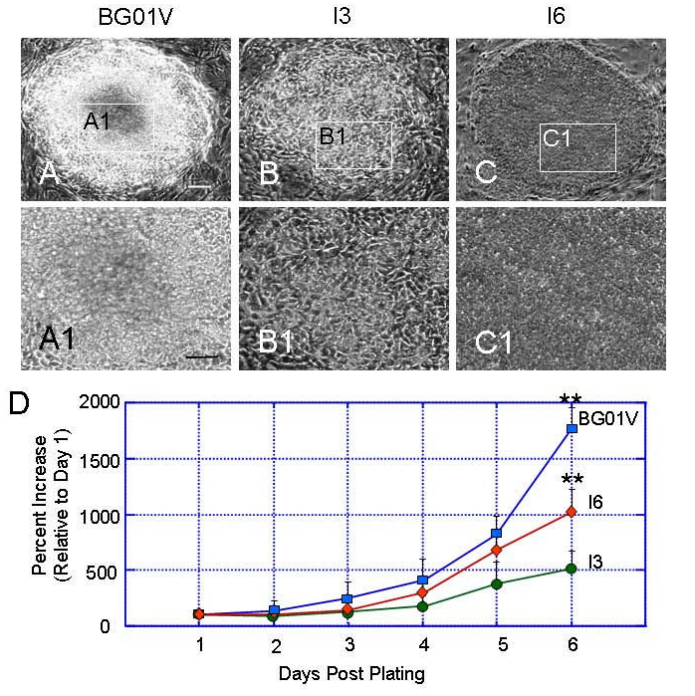
**(A-C) Morphology of undifferentiated colonies of hESC lines I3, I6, and BG01V**. Phase contrast photographs of hESC colonies cultured on mouse embryonic fibroblast feeders for 4 days. The boxes in the center of the colonies indicate areas shown in A1-C1. (A1-C1) High magnification of the center regions of the colonies from each cell line showing distinct morphologies. The colonies of BG01V and I6 lines exhibit highly compact cells with rather vague borders (A1 and C1), while I3 colonies have a mosaic appearance with loosely packed cells (B1). Bars in A and A1 = 100 μm. (D) Three human embryonic stem cell lines display distinct growth characteristics under the same culture conditions. The growth curves show significant differences in percent increase in the number of cells at day 6 in culture between the three cell lines. I3 cells grow slower than the other two cell lines and have a tendency to differentiate. Therefore, it is more difficult to maintain the I3 cell line in an undifferentiated state. In contrast, the I6 and BG01V cell lines grow faster and are passaged 2–3 days earlier than the I3 cells. Statistical differences for percent increases in cell numbers at 6 days between the BG01V or I6 and I3 are significant ** p < 0.01.

Undifferentiated hESC lines BG01V, I3 and I6 that had been maintained on mitomycin C-treated mouse embryonic fibroblasts (MEF) for 4–5 days displayed distinct cell colony morphologies. Cells in the center regions of colonies exhibited prominent nucleoli and a high nucleus to cytoplasm ratio. Cells from BG01V (passage 22) and I6 (passage 50) lines were highly compact with rather vague borders (Figure 1A and 1A1, 1C and 1C1). Their colonies exhibited a round and sharp edge separating the hESC from surrounding feeder cells. In contrast, colonies of I3 cell line (passage 58) exhibited a mosaic appearance with loosely packed cells (Figure 1B and 1B1). This observation is consistent with previous descriptions [21,22].

### Differences in growth curves between undifferentiated hESC lines

To examine potential differences in the ability to self-renew between the three hESC lines, the percent increase in cell numbers relative to cell numbers at day 1 were calculated up to 6 days (Figure 1D). The relative growth was determined, followed by the plating of approxmately 10^5 ^cells on a MEF feeder, and trypsinized cells were counted using a Cedex Analysis System. The differences in the cell number between each cell lines should represent the differences in relative growth of hESCs since the fibroblast feeder cells were mitomycin C-treated. The growth curves in Figure 1D show significant differences in percent increase in the number of cells at day 6 in culture between the three cell lines. I6 and BG01V cell colonies reached an average size of 300–400 cells for splitting at 5–6 days after passaging. In contrast, I3 cells grew slower than the other two cell lines and were not ready to passage until culture day 8. It appeared more difficult to maintain the I3 cell line in an undifferentiated state since it had more tendency to differentiate.

### MPSS expression analysis of three human ESC lines

**Figure 2:**
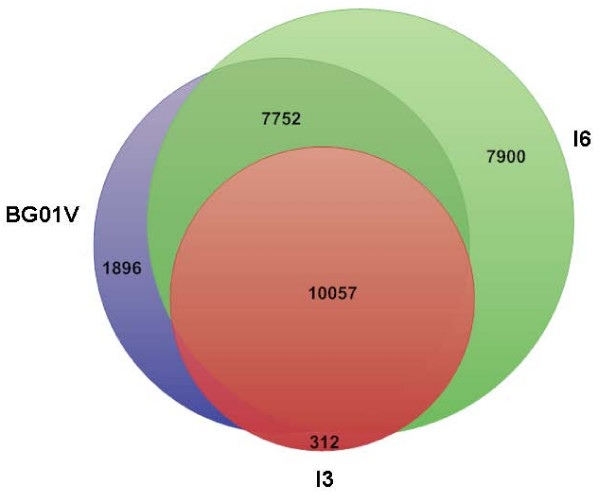
**Digital gene expression of the three undifferentiated hESC lines**. It shows that around 92% (10057 out of 10945) of the genes expressed in the I3 cells were also expressed in the BG01V and I6 cells. The large overlap in genes expressed among the 3 hESC lines suggests the presence of a relatively stable core "stemness" transcriptome.

After full annotation of over one million sequenced signatures from each ESC line, there were 28, 071 human UniGene clusters detected by MPSS in the three human undifferentiated ESC lines (BGO1V, I3 and I6). I6 expressed the most genes (25,862) and I3 expressed the fewest genes (10, 945); BGO1V cells expressed 19, 858. Although the differences in numbers of total expressed gene numbers among the three ESC lines was significant, the number of core transcriptome expression of the three hESC lines were substantial: 95.7% (10479/10945) and 93.3% (10210/10945) of I3 genes were expressed in I6 and BGO1V, respectively, and 92% (10,057 out of 10, 945) genes of I3 were co-expressed in the other two lines (Figure 2). These findings indicate that different hESC lines retain core transcriptome features of embryonic "stemness".

### All undifferentiated hESC lines express pluripotency markers, but their gene expression levels are variable

**Figure 3:**
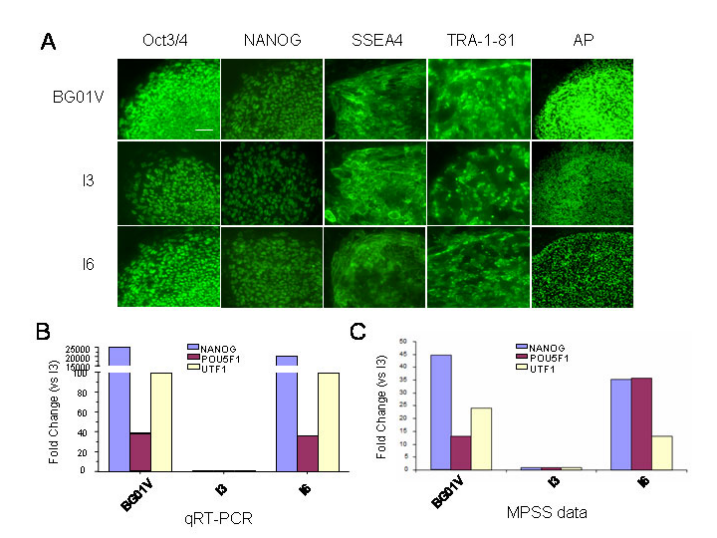
**Differences in relative expression levels of undifferentiated cell ("stemness") markers among the three undifferentiated hESC lines**. (A) Immunofluorescent staining of hESC colonies shows an expression of Oct3/4, NANOG, SSEA4, TRA-1-81 and alkaline phosphatase (AP) activity in all three cell lines. Bar = 100 μm. (B) Quantitative RT-PCR analysis of the relative expression levels of "stemness" genes (NANOG, POU5F1, and UTF1) in three undifferentiated hES cells. The Y-axis plots the fold change of the undifferentiated cell lines in comparison to the undifferentiated I3 cell lines. The expression level of the undifferentiated genes implicates that the I3 hES cells express much less "stemness" (undifferentiated) genes than the I6 and BG01V cell lines. (C) MPSS analysis shows the consistency of the expression levels of the undifferentiated genes (Nanog, POU5F1, and UTF1) compared with the results of the qRT-PCR analysis (B).

The undifferentiated state of human ES cells was first characterized by immunocytochemistry. All the three hESC lines expressed glycolipid antigens such as stage-specific embryonic antigen SSEA-4, tumor rejection antigen TRA-1-81, and transcription factors Oct-3/4 and NANOG as described previously [23]. All cell lines also exhibited alkaline phosphatase (AP) activity (Figure 3A). To further study the quantitative expression of pluripotency markers, quantitative RT-PCR and MPSS were used to assay the expression of a series of genes in each of the three undifferentiated hESC lines (Figure 3B) and in their differentiated EB. We cross validated the results of quantitative RT-PCR and MPSS analysis of the expression levels of undifferentiated hESC marker genes (NANOG, POU5F1, and UTF1) in the three hESC lines over time.

The Y-axis plots the fold change of the undifferentiated I6 and BGO1V cell lines in comparison to the undifferentiated I3 cell line. The expression level of the undifferentiated genes implicates that the I3 hES cells express much lower levels of "stemness" (undifferentiated) genes compared to the I6 and BG01V cell lines (Figure 3B and 3C).

### Differences in BrdU incorporation between undifferentiated I3, I6 and BG01V cells

**Figure 4:**
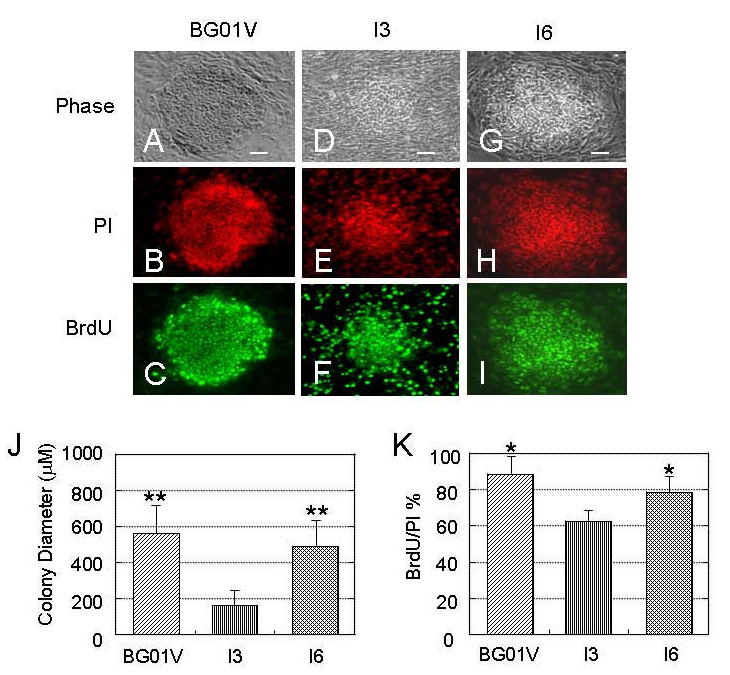
**Differences in the proliferation rate between the undifferentiated I3, I6 and BG01V cells**. All three cell lines were maintained on the MEF for 4 days post-passaging under the same culture conditions. A four hour BrdU pulse shows significant differences in the proliferation index which is defined as the percentage of BrdU+ nuclei among the total number of propidium iodide (PI)+ cells.(A-I) Images of the three cell line colonies (A, D, G) immunostained for BrdU incorporation (C, F, I) and counterstained with PI (B, E, H). Bars in A, D and G = 100 μm. (J) Bar plot summarizing the differences in diameters of the colonies between the three cell lines. Values are expressed as a percent of the total number of cells (mean ± SEM; BG01V- 554 ± 187 μm, I3 – 184 ± 75 μm, and I6 – 488 ± 165 μm. Sizes of colonies derived from the BG01V or I6 cells are significantly greater than those derived from the I3 cells. ** p < 0.01. (K) Bar plot summarizes the differences in the BrdU incorporation levels between three cell lines. The BG01V cells maintained on MEF at 4 days post-passaging exhibit the highest BrdU incorporation levels and the I3 exhibited the lowest levels. Values are expressed as the ratio of BrdU+ cells to the total number of cells (PI stained) (mean ± SEM). Statistical differences for colony diameters or BrdU/PI % between the I6 or BG01V and the I3 cells are significant * p < 0.05.

To examine the potential difference in the ability to proliferate between different the hESC lines, bromodeoxyuridine (BrdU) incorporation assays were performed in colonies of I3, I6 and BG01V cells (Figure 4A, D, G). hESC maintained on the MEF for 4 days under the same conditions were incubated with BrdU for 4 h before being processed for BrdU immunocytochemistry (Figure 4C, F, I). Cells were counterstained with PI which stained nuclei of all cells (Figure 4B, E, H). We indexed proliferation for each cell line by quantifying the proportion of BrdU+ cells versus the total number of cells (PI-stained cells). At 4 days post-passaging, all three cell lines showed active DNA synthesis, but the I3 cell line exhibited significantly smaller colonies and a lower proliferation rate compared to the other two cell lines (Figure 4J). Unlike the I6 and BG01V cell colonies within which BrdU+ cells were confined, some BrdU+ cells were scattered outside of the I3 colonies, although the majority of BrdU+ cells were located within the colonies (Figure 4F). The scattered BrdU+ cells may represent individual proliferative I3 cells on the MEF layer before forming a colony, indicating much slower proliferation in the I3 cells compared to the I6 and BG01V cells. Cell counting showed differences in BrdU incorporation between the three cell lines. BG01V cells exhibited highest BrdU incorporation levels (88% ± 12%), while I3 cells had the lowest levels (62% ± 3.1%) (Figure 4K). The proliferation rate in I6 cells was 78% ± 9.2%. The difference between three cell lines in BrdU incorporation was consistent with the sizes (diameters) of the colonies. At 4 days in culture I3 cells exhibited smaller colonies with a lower proliferation index compared to the BG01V and I6 cells. Colonies of the I6 and BG01V cells exhibited higher numbers of active DNA synthesizing cells compared to the I3 cells.

### Embryoid body formation and growth rate vary among the three hESC lines

**Figure 5:**
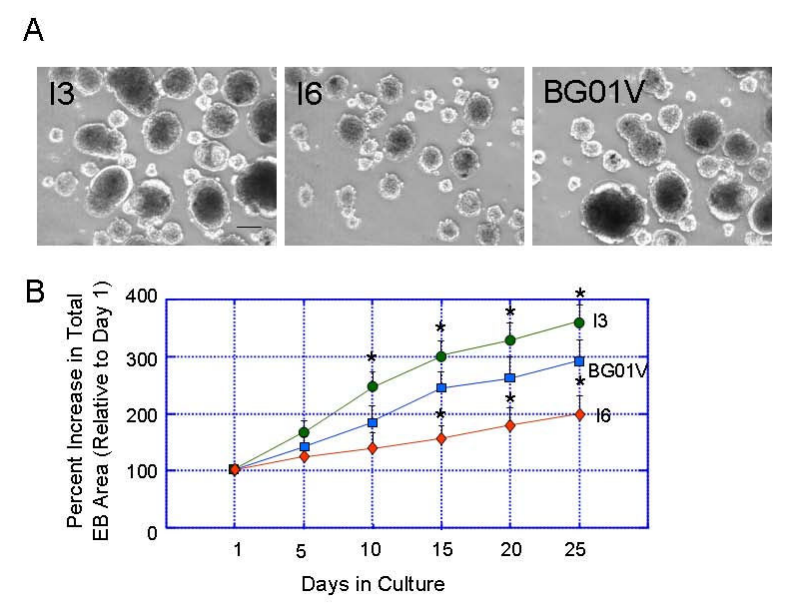
**Differences in the embryoid body (EB) growth rates between the hESC lines I3, I6 and BG01V**. hESC colonies were removed from feeder monolayers and grown in low attachment dishes. (A) Phase contrast images of the EBs in the three cell lines cultured in suspension for 10 days. (B) A plot showing differences in the EB growth rate between three cell lines. The growth rate of EBs was calculated by the increase of EB size (total EB area) in each 5 days from day 1 though 25. Values are expressed as percent increase in total areas of EBs (mean ± SEM). Statistical differences for EB growth rates after 10 days in culture between I3 and I6 or BG01V are significant * p < 0.05. Bar in upper panel = 200 μm.

EB represents a unique tool to investigate *in vitro *differentiation processes of hESCs. Colonies of the three ESC lines were removed from feeder cells and grown as cell aggregates in a suspension in low attachment dishes without basic fibroblast growth factor. The growth rate of the EBs was calculated by the increase of EB size (total EB area) in every 5 days period from days 1 though 25 (Figure 5A). The percent increase in size of the cell spheroids was significantly different in the EBs from the three different hESC lines after 10 days in the suspension culture (Figure 5B). The relative EB growth was greater in the I3 and BG01V cells compared to the I6 cells.

### All three hESC lines are able to differentiate into cells expressing markers of all three germ layers and neural cells

**Figure 6:**
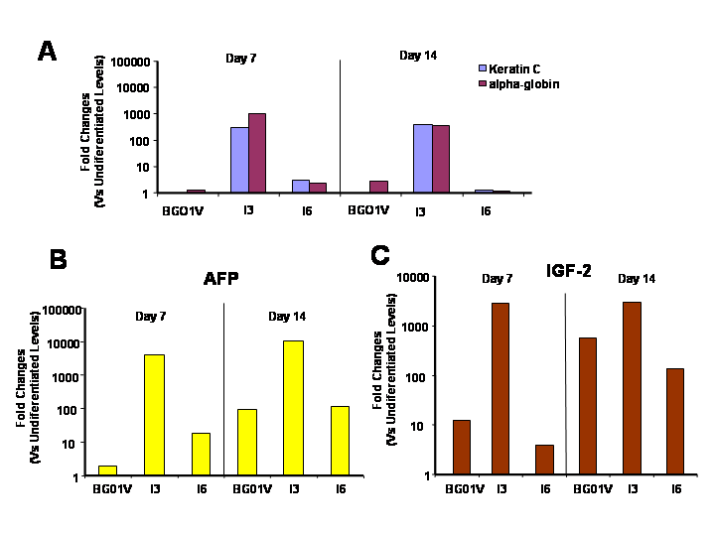
**Differences in expression of three germ layer markers in the embryoid bodies (EBs) derived from three hES cell lines BG01V, I3 and I6**. (A) Quantitative RT-PCR analysis of expression of keratin C (ectoderm marker) and alpha-Globin (mesoderm marker) at 7 and 14 days in the EBs derived from the three cell lines. The Y-axis plots the fold changes (log scale) in expression levels of the two genes for each cell line when compared to its own undifferentiated levels at Day 0. The I3-derived EBs cultured at days 7 and 14 expressed markedly higher levels of the two genes compared to I6 and BG01V-derived EBs. (B and C) Higher expression of endoderm marker AFP and mesoderm marker IGF-2 gene expression in the I3 than in the BG01V and I6 cell lines revealed by MPSS. Quantitative MPSS analysis of the AFP and IGF-2 in the EBs derived from the hES cell lines BG01V, I3 and I6. The Y-axis plots the fold change (log scale) in expression levels for each cell line when compared to its own undifferentiated cells (Day 0).

EB formation is a model of *in vitro *embryogenesis in which all three primary embryonic germ cell lineages are generated [24]. To examine differences in the gene expression of the three germ layer markers in EBs derived from three ESC lines, we assessed the expression of keratin C (ectoderm) and alpha-globin (mesoderm) by qRT-PCR (Figure 6A), and expression of alpha FP (endoderm) and IGF2 (mesoderm) by MPSS (Figure 6B, C). The fold changes (log scale) in the expression levels of keratin C and alpha-Globin for each cell line relative to its own undifferentiated levels showed that the I3 cell line expressed markedly higher levels of these two genes at 7 and 14 days when compared to the I6 and BG01V cell lines (Figure 6A). MPSS results showed that the I3 cell line expressed higher levels of both Alpha FP and IGF-2 than the I6 and BG01V cell lines (Figure 6B, C).

**Figure 7:**
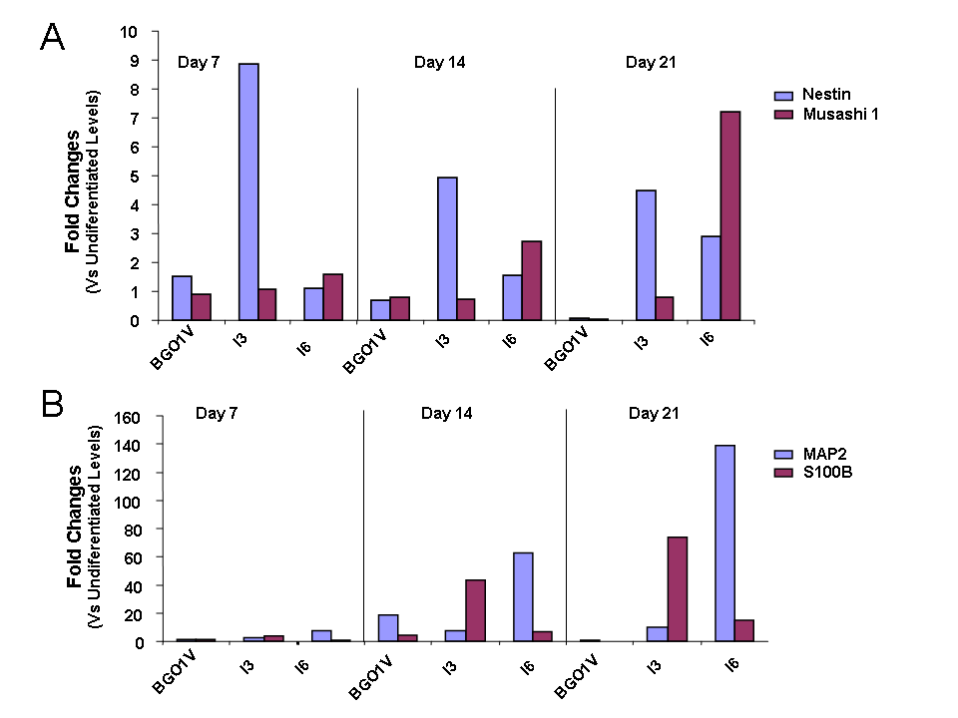
**Quantitative RT-PCR analysis shows differences in expression levels of neural cell lineage-specific genes between three hES cell lines**. The Y-axis plots the fold change for each cell line when compared to its own undifferentiated cells (Day 0). (A) shows a comparison of expression levels of the neural progenitor-specific genes at days 7, 14 and 21 of differentiation. The I3 and I6 cells exhibited higher levels of Nestin and Musashi 1 gene expression than BG01V cells. (B) shows a comparison of the expression levels of the neuronal (MAP2)- and glial (S100B)-specific genes at days 7, 14 and 21 of differentiation. Both of the MAP2 and S100B gene expressions were up-regulated in the I3 and I6 cells.

Since a primitive neural stem cell stage can be acquired though a default mechanism [25], we assessed the expression levels of the neural markers, Nestin and Musashi 1 (neural progenitor), MAP2 (mature neurons) and S100B (astrocytes) by qRT-PCR and MPSS (Figure 7). qRT-PCR analysis showed differences in expression levels of these genes between the three hES cell lines. The I3 and I6 cells expressed higher levels of neural progenitor-specific genes (Nestin and Musashi) (Figure 7A), as well as neuronal gene (MAP2) and glial gene (S100B) (Figure 7B) than BG01V cells. Both MAP2 and S100B genes were upgraded from day 7 to Day 21 in culture in the I3 and I6 cells.

### Differences in expression of neural phenotypes and genes in directed neural differentiation between three hESC lines

**Figure 8:**
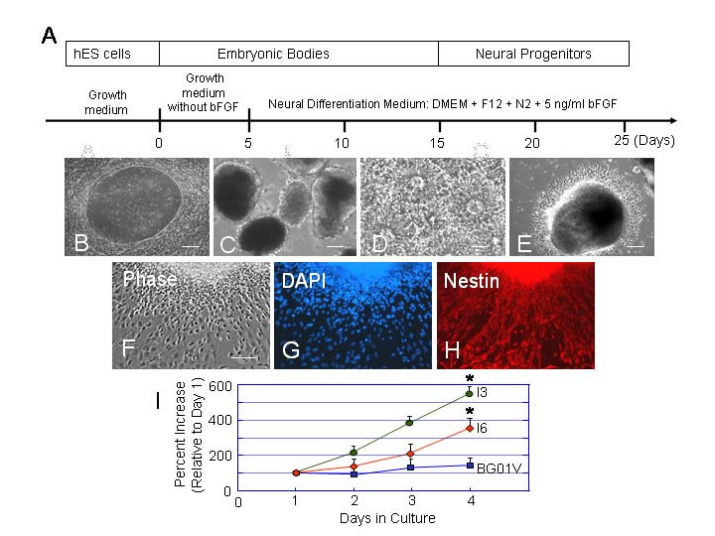
**The comparison of the neural progenitor derivation from the I3, I6 and BG01V hESC lines over time under the same neural differentiation-promoting condition**. (A) The protocol used to direct hESCs into neural cell lineages. (B-E) Phase contrast images show the progression of the neural induction of hESCs in which the hESC colonies (B) were removed from the MEF feeders and grown into floating aggregates or embryoid bodies (EBs) (C). Neural rosettes (D) were induced by the neural differentiation medium. New cells constantly generated and migrated radially away from the center of the EB and formed a rim of cells (E). Bars in B-E = 100 μm. (F-H) Neural progenitors derived from the I3 cell line. Phase contrast image of I3-differentiated cells (F) were immunostained for nestin (H) and nuclear counterstained with DAPI (G). (I) The plot shows significant differences in the average percent increase in the number of nestin+ cells differentiated from the three cell lines. Values are expressed as percent increase in Nestin+ neural progenitors (mean ± SEM). Statistical analysis shows that the percent increase in number of Nestin+ cells generated from the I3 or I6 cells is significantly greater than that generated from BG01V cells, * p < 0.05. Bar in upper panel = 200 μm.

**Figure 9:**
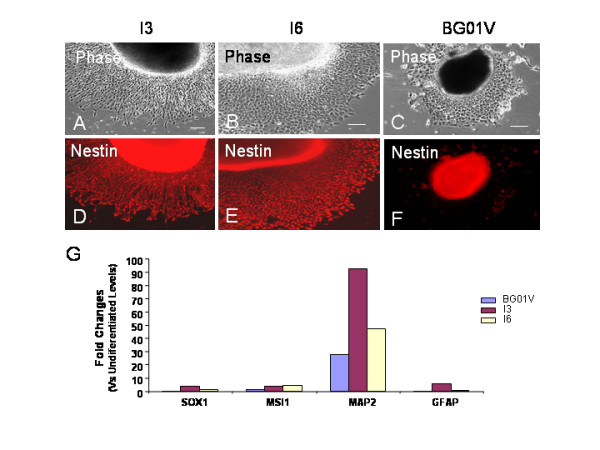
**Difference in directed neural differentiation between hESC lines I3, I6 and BG01V**. Two days after transferring EBs to a poly-D-lysine/laminin substrate, parallel immunocytochemistry and quantitative RT-PCR were performed in hESC-derived cell populations. Immunofluorescent staining for Nestin (A-F) shows that both the I3 and the I6 cells differentiate into the enriched Nestin^+ ^neural progenitors while BG01V cells barely generate Nestin^+ ^cells. Bars in A-C = 100 μm. (G) qRT-PCR analysis of the gene expression of the neural progenitor markers SOX1 and MSI1, mature neuronal marker MAP2, and astrocyte marker GFAP among the three cell lines at day 17 of differentiation. The Y-axis represents the fold changes of the gene expression for each cell line when compared to its own undifferentiated levels at day 0. The relative levels of these genes expressed by the I3 and I6 cells are higher than those expressed by BG01V cells.

Differences in pluripotency between the three cell lines were also examined during the directed neural differentiation. To test the possible differences we used a reliable step-wise differentiation protocol (Figure 8A) which generated highly pure neural progenitors from the I3 and I6 cells [18]. The hESC colonies were removed from MEF feeders and cultured in suspension in low attachment dishes with hESC medium without bFGF for 5 days (Figure 8B, C). Differentiating EBs were transferred into the neural differentiation medium for 10 days. At days 15–17 of differentiation, EBs were plated on poly-D-lysine/laminin-coated 35 mm dishes. Neural rosettes were visualized after plating of the EBs on the substrate (Figure 8D). Neuroectodermal cells in neural rosettes were stained for Sox1 and Nestin (not shown), and further differentiated into neural progenitors and their progeny (Figure 8E). Under the same neural differentiation-promoting condition, we quantified the percentage of hESC-differentiated neural progenitors by immunostaining for Nestin, together with nuclear DAPI counterstaining (Figure 8F, G, H). Nestin+ cells were manually counted and were expressed as a percentage of the total DAPI labeled cells (Figure 8I). A significant difference was found in the percentage increase in number of Nestin+ cells differentiated from the three cell lines after 3 days post plating (Figure 9A–F). The percentage increase in Nestin+ cells generated from the I3 cells was greater than that of the I6 cells. The BG01V generated undetectable or an insignificant number of neural progenitors which were lightly scattered among differentiated cells. In addition, we tracked the appearance of neural rosettes during neural differentiation and found that the I3-derived rosettes were generated 5–9 days earlier than the I6-derived neural rosettes. The BG01V-derived neural rosettes were barely detected.

To assess differences in the gene expression during neural differentiation between the three cell lines, total RNA was harvested from the EBs at 2 days post plating on poly-D-lysine/laminin substrates. The qRT-PCR analysis of the expression levels for the neural progenitor markers SOX1 and MSI1, the mature neuron marker MAP2, and the astrocyte marker GFAP showed that the all these genes were strongly expressed in the I3 cells (Figure 9G). The Y-axis plots the fold changes of gene expression for each cell line when compared to its own undifferentiated levels at Day 0. The high levels of expression for the neural progenitor genes SOX1 and MSI1 in the I3 and I6 cells were consistent with their high expression of nestin immunoreactivity (Figure 9A, B).

## Discussion

In the present study, we demonstrated that although all the three hESC lines I3, I6 and BG01V can maintain their ability to proliferate and give rise to the progeny of the three embryonic germ layers, their self-renewal and differentiation capabilities are variable. The overall gene expression profiles of the three lines were similar; however, in most cases, the relative abundance of expression of the same "stemness" and differentiation genes were highly variable between the cell lines. We also found that under the same neural induction conditions, the ability of each of the three lines to differentiate into neural progenitors was also distinct. Previous studies that compared hESC lines focused on the expression of pluripotency and the differentiation marker genes [9,10,12,26]. In the present study, in addition to the variable gene expression in undifferentiated and EB differentiated states, we detected a profound variation in the cell growth rate, BrdU incorporation and the directed neural differentiation.

The work presented here is part of continuing efforts to develop a database of the properties and behaviors of different hESC lines and to understand the similarities and differences between individual hES cell lines by side-by-side comparison. This comparative analysis of individually-derived hESC lines is critical, because the properties and behavior of each line are uniquely shaped by their histories. It has become clear that different derivations produce hESC lines that are similar with regards to "stemness", but with inherent differences in gene expression, methylation status, X chromosome inactivation, rate of self-renewal and the ability to differentiate [6,26,27]. More importantly, the behavior of cells and their overall state changes as culture conditions and the stress they are subjected to is altered, and permanent genomic changes frequently occur as passage numbers increase [28-30]. Variability in genetic, environmental and methodological factors has led to a great difficulty in comparing results of studies of the hESC lines among laboratories.

In this study, our side-by-side comparison between the three hESC lines was made under the same culture conditions in an effort to minimize the influences of environmental and methodological factors. The differences we found between the three cell lines may be due to the genetic variation and epigenetically inherited alterations from previous culture history. Lines I3 and I6 were derived in the same laboratory but differ in sex [15]. BG01V is a variant cell line with an abnormal karyotype [17]. Another factor that may contribute to the cell line differences is the variation in passage number between the three cell lines. It is more challenging to directly compare differences in directed differentiation between different human ES cell lines because the differentiation protocols most likely are cell line-specific. Previous study has shown that the reliable dopaminergic differentiation was induced by co-culture with the mouse stromal cell line PA6 [16]. However, in the present study, under culture conditions that favored neural differentiation of the I3 and I6 cells, the BG01V barely produced neural progenitors and expressed much fewer neural-specific genes compared to I3 and I6 cells. The inconsistency in BG01V cell neural differentiation data between Zeng's and our studies points out that each hES cell line needs an optimized protocol for a specific phenotype differentiation.

hESC lines have a great potential to provide new research tools that support clinical applications. The frequency of non-obvious changes in the hESC behavior and potency is of great concern for the future of cell replacement therapies. Physicians who transplant hESC-derived cells into patients must be in confident as to the safety and stability of the cells they use. Thus it is necessary not only to establish a set of characterization tests which are sensitive enough to detect small but harmful changes, but these tests must also be simple and inexpensive enough to be used routinely. The comparison made in this study also shows that the individually derived hESC lines from different laboratories are variable to various extents. Therefore reference standards, such as cell lines that provide consistent, predictable results and are not difficult to culture are needed. We believe that the database of hESC characterization data and standard reference materials will permit the research community to readily monitor and compare hESC lines.

## Conclusion

Our side-by-side comparison confirms the general finding that hESC lines share the properties of self-renewal, expression of "stemness" and pluripotency markers and the ability to differentiate, but many differences remain between cell lines. These differences include the ability to maintain an undifferentiated state, to self-renew, and to differentiate. In addition to inherited variation in the sex, stage, quality and genetic background of embryos used for hESC line derivation, these differences may be associated with derivation methods and changes acquired during passaging in culture. To this end, it is important to set up standards shared by multiple laboratories for routine analysis of undifferentiated state ("stemness"), identity, stability, pluripotency and sterility of hESC lines.

## Authors' contributions

WM, TT, MR and MM were primarily responsible for the data analysis and writing or editing the manuscript. YR reviewed and edited manuscript. XX performed MPSS analysis. TT, ED and JS carried out the hES cell cultures and immunocytochemistry. All authors read and approved the final manuscript.
